# Efficacy of Medicated Thread Moxibustion of Zhuang Medicine on Skin Lesions in Eczema Rats Based on p38/NF-κB and JAK1-STAT6 Pathways

**DOI:** 10.1155/mi/9978298

**Published:** 2025-09-02

**Authors:** Liangbing Wu, Jian Dai, Yongzheng Wei, Quanrui Jiang, Renkun Huang, Yahui Wang, Xingling Chen, Jiandie Chen, Jinhua Yao, Zhenjie Qiu, Panyu Jiang, Yanyang Zhao, Bingyi Zheng, Wei Lu

**Affiliations:** Faculty of Acupuncture, Moxibustion and Tuina, Guangxi University of Chinese Medicine, Nanning 530001, Guangxi, China

**Keywords:** eczema, inflammation, JAK1-STAT6, medicated thread moxibustion of Zhuang medicine, p38/NF-κB, skin lesion

## Abstract

**Objective:** Eczema is a common inflammatory skin disease that severely affects patients' daily life and work, necessitating effective intervention. Medicated thread moxibustion of Zhuang medicine (MTMZM), an integral part of Chinese medicine, is also a component of complementary and alternative medicine, demonstrating promising therapeutic effects. However, its mechanism in treating eczema remains unknown. Therefore, this study investigated the efficacy and mechanism of MTMZM on skin lesions and p38/NF-κB and JAK1-STAT6 pathway in eczema rats.

**Methods:** Forty-eight male Sprague-Dawley (SD) rats were randomly divided. Nine of them were assigned to the normal group, while the remaining 39 rats were selected for the subsequent eczema model establishment process. In total, 7% DNCB acetone olive oil solution was used to establish eczema model. Successful modeling rats were randomly divided into three groups with 13 rats each: model group, western medicine group (WM group), and MTMZM group. Normal group and model group received no treatment. MTMZM group received MTMZM treatment on the Ashi point (skin lesions in eczema) and WM group received positive drug Pevisone cream. The eczema severity index (ESI) in rats was scored before intervention and during the first and second weeks of intervention. After intervention, samples were taken from rats' back lesions (taking normal skin in the same area from normal group). After sampling, the skin thickness difference (STD) with normal skin and diseased skin lesions was measured. HE staining was used to observe the tissue morphology of skin lesions. Western blot was used to detect JAK1, p-JAK1, STAT6, p-STAT6, NF-κB p65, p-NF-κB p65, and p-p38 protein content in skin lesions; the serum content of interleukin (IL)-1β, tumor necrosis factor-α (TNF-α), and IL-4 were detected by ELISA.

**Results:** (1) Compared with normal group, model group showed dermal necrosis and inflammatory cell infiltration under light microscopy. The ESI and STD increased (*p*  < 0.05). JAK1, p-JAK1, STAT6, p-STAT6, NF-κB p65, p-NF-κB p65, and p-p38 protein content in skin lesion increased(*p*  < 0.05). The serum content IL-1β, TNF-α, and IL-4 increased (*p*  < 0.05). (2) Compared with model group, MTMZM group and WM group showed significant improvement in pathological changes. The ESI and STD decreased (*p*  < 0.05). NF-κB p65, p-NF-κB p65, p-p38, JAK1, p-JAK1, STAT6, and p-STAT6 content (*p*  < 0.05) decreased. The serum content IL-1β, TNF-α, and IL-4 decreased (*p*  < 0.05). (3) Compared with WM group, MTMZM group showed visible neovascularization under light microscopy. The ESI and STD decreased (*p* < 0.05). There was no significant difference in p-p38, p-NF-κB p65, JAK1, p-JAK1, STAT6, and p-STAT6 content (*p*  > 0.05), as well as in IL-4 content (*p*  > 0.05). The serum content IL-1β and TNF-α increased (*p*  < 0.05).

**Conclusion:** MTMZM can effectively relieve eczema skin lesions, which may be related to the inhibition of p38/NF-κB and JAK1-STAT6 pathways.

## 1. Introduction

Eczema, an inflammatory skin disorder, is characterized by diverse skin lesions exhibiting symmetrical distribution, exudation, pruritus, and a tendency for recurrent attacks, often leading to chronicity [1]. The prevalence of eczema among adults was 5.4%–5.6% [2]. Despite the multitude of therapeutic approaches developed in modern medicine, the prolonged use of conventional therapies, such as hormone-based medications, often yields less than optimal outcomes and is accompanied by adverse side effects, thereby driving researchers to continually pursue safer and more efficacious treatment regimens [3]. Medicated thread moxibustion of Zhuang medicine (MTMZM), a unique external therapy of Zhuang medicine, is a national intangible cultural heritage and one of the major moxibustion methods recommended by China National Planning Textbook for Higher Education [4]. This therapy uses ramie threads soaked in Zhuang herbal medicinal materials for a certain period of time, then, ignites and directly touch specific acupoints or parts of the patient's body surface to achieve the purpose of treating diseases [5].

Our research team found that MTMZM has good efficacy in treating eczema, but its mechanism needs to be more studied [6, 7]. The cause and pathogenesis of eczema remain unclear, which may be closely related to environmental, genetic, immune, infection, mental, and other factors [8]. Inflammation stands as a pivotal factor in the pathogenesis and progression of eczema [9], and further investigation is warranted to elucidate the specific influence of MTMZM on this intricate process. In inflammatory, classic signaling pathways, such as p38/NF-κB and JAK1/STAT6 are activated, and their products, such as interleukin (IL)-1β, tumor necrosis factor-α (TNF-α), and IL-4 increase, thereby promoting the aggregation, activation and effects of inflammatory cells, and ultimately aggravating the inflammatory response [10, 11]. To sum up, we hypothesized that the mechanism of MTMZM in treating eczema may be related to the inhibition of p38/NF-κB and JAK1-STAT6 signaling pathways. The eczema rat model is established to observe and assess the pre- and posttreatment variations in clinical index scores, morphological characteristics, proteins involved in signal transduction pathways, and levels of inflammatory cytokines following the administration of MTMZM.

This research aims not only to provide empirical support for the scientific principles of MTMZM and reveal its novel mechanism of inhibiting inflammatory response, but also to offer new insights and strategies for the clinical treatment of eczema and other inflammatory skin diseases. Furthermore, it is expected to promote the deep integration of traditional medicine and modern medicine, ultimately providing patients with safer and more effective treatment options.

## 2. Materials and Methods

### 2.1. Experimental Animals and Grouping

**Figure 1:**
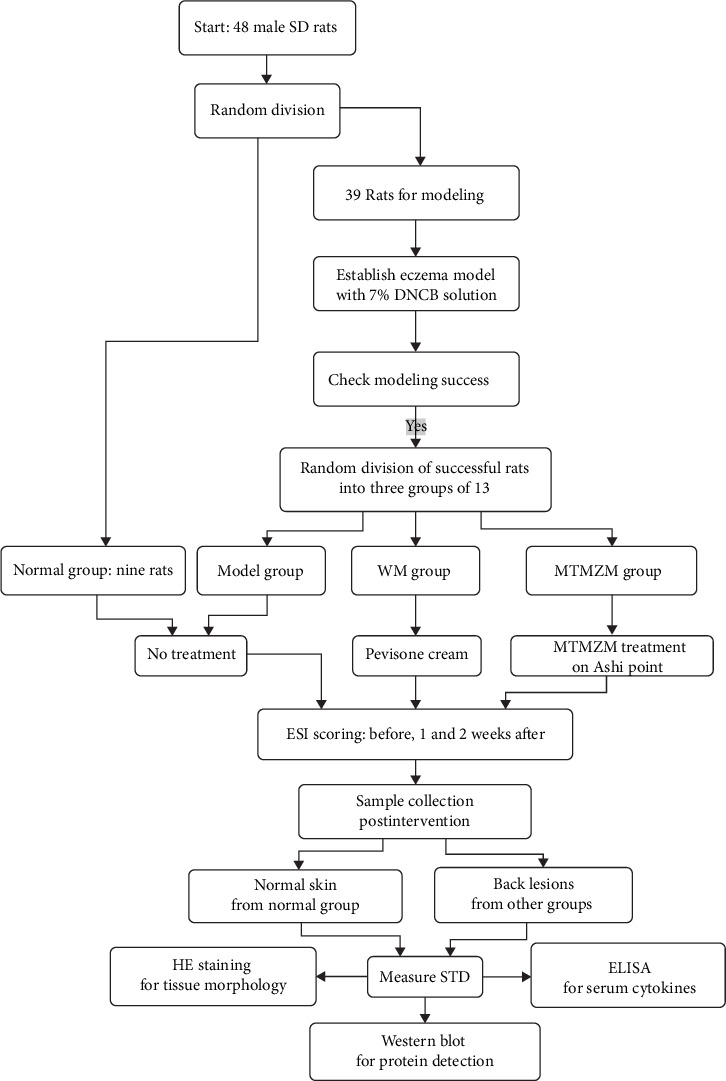
Experimental process.

A total of 48 specific pathogen-free (SPF) grade healthy male Sprague-Dawley (SD) rats (9–10 weeks old, weighing 220–250 g) were acquired from Hunan SJA Laboratory Animal Co., Ltd (SCXK (XIANG) 2019-0004), and were acclimated in SPF Animal Laboratory, Science Laboratory Center, Guangxi University of Chinese Medicine (temperature: 20–25°C, relative humidity: 40%–79%, and light–dark cycle: 8:00–20:00 in daytime light) for 1 week with regular feeding of standard grain and free access to food and water. This study was approved by the Experimental Animal Ethic Committee of Guangxi University of Chinese Medicine, Nanning, China (Approval Number DW20210506-071). The disposal of animals in the experiment strictly complies with the relevant provisions of the Guidelines on the Good Treatment of Laboratory Animals issued by the Ministry of Science and Technology of the People's Republic of China in 2006. All rats were divided into two groups by random number table methods: normal group with nine rats and model group with 39 rats. After modeling, the rats were then randomly divided into three groups of 13 rats each: model group, Western medicine (WM) group, and MTMZM group. The experimental process is shown in Figure 1.

### 2.2. Main Reagents and Instruments

#### 2.2.1. Main Reagents

1-Chloro-2,4-dinitrobenzene (Lot Number: 237329, Sigma–Aldrich), acetone (Lot Number: 2021109, Chengdo Kelong Chemical Co., Ltd), olive oil (AGRIC), Pevisone cream (Lot Number: H20000454, Xian Janssen Pharmaceutical Ltd.), HE staining kit (Lot Number: C0105M, Shanghai Beyotime Biotechnology Co., Ltd), protein phosphatase inhibitors 3 (Lot Number: P0044, Sigma), protease inhibitor (Lot Number: P8640, Sigma), BCA protein assay kit (Lot Number: 23225, ThermoFisher), 4%–15% protein gradient gel (Lot Number: 5671085, BIO-RAD), anti-JAK1 antibody (Lot Number: AF5012, AFFINITY), p-JAK1 antibody (Lot Number: AF2012, AFFINITY), anti-STAT6 antibody (Lot Number: AF6302, AFFINITY), p-STAT6 antibody (Lot Number: AF3301, AFFINITY), β-tubulin antibody (Lot Number: 2146s, CST), p-P38 antibody (Lot Number: AF2006, AFFINITY), NF-κB p65 antibody (Lot Number: AF5006, AFFINITY), p-NF-κB p65 antibody (Lot Number: AF2006, AFFINITY), GAPDH antibody (Lot Number: AF7021, AFFINITY), rat IL-1β kit (Lot Number: ED-30206, Xiamen Lunchangshuo Biotechnology Co., Ltd), and rat IL-4 ELISA Kit (Lot Number: ED-30217, Xiamen Lunchangshuo Biotechnology Co., Ltd).

#### 2.2.2. Main Instruments

Desktop High-speed Refrigerated Microcentrifuge (Model Number: D3024R, DLAB Scientific Co., Ltd), Microplate reader (Model No: RT-6100, Rayto), Vertical Electrophoresis System (Model Number: 1658001, BIO-RAD), Trans-Blot (Model Number: 1703930, BIO-RAD), Western Transfer (Model Number: 1704273, BIO-RAD), Gel Imaging system (Model Number: 12003254, BIO-RAD), and Upright White Light Photographic Microscope (Model Number: Eclipse Ci-L, Nikon).

### 2.3. Animal Model

The eczema model was established according to Fujii et al. [12]. 1-Chloro-2,4-dinitrobenzene (dinitrochloro benzene [DNCB]) is a chemical substance that can easily cause allergic reactions. It can sensitize local skin and cause inflammatory reactions after being stimulated. A 7% DNCB acetone olive oil solution (DNCB solution) was prepared by using a mixture of 4:1 acetone and olive oil with 1-chloro-2,4-dinitrobenzene.

After 1 week of routine adaptive feeding of rats, the abdominal Skin A and back Skin B of each rat were shaved. The area of A was 2 cm × 2 cm, and the area of B was 4 cm × 4 cm. Normal group was not sensitized or stimulated. Rats in the remaining groups were sensitized by applying 100 μL of 7% DNCB acetone solution to the skin at Area A using a pipette. After sensitization, rats showed severe itching and frequent scratching and rolling behaviors for about 2 h. One week later, 200 μL of 7% DNCB acetone solution was applied to Site B for stimulation. The rats were stimulated once every 5 days. During each stimulation, we found that the rats showed severe itching with frequent scratching and rolling behaviors. The skin at Site B gradually appeared erythema, papules, edema, scratches, and desquamation. The skin lesions were recorded and scored after each stimulation. After six times stimulations, erythema, papules, scabs, and exudation appeared, indicating successful model preparation.

### 2.4. Intervention

#### 2.4.1. Drug Treatment

Pevisone cream is currently recognized as an effective drug for the treatment of eczema [13]. After modeling, WM group received Pevisone cream (generic name: triamcinolone acetonide and econazole nitrate cream) intervention, 250 mg per time, once a day, evenly applied to eczema lesions of B area for 2 weeks, 1 week as one course of treatment.

#### 2.4.2. Manipulation

Preparation of medicated thread is as follows [14]:

Take 10 g of Ru Xiang (frankincense, *Boswellia sacra*), 10 g of Mo Yao (myrrh, *Commiphora myrrha*), 10 g of Tan Xiang (sandalwood, *Santalum album*), 10 g of Mu Xiang (costus root, *Saussurea costus*), 10 g of Tao Ren (peach kernel, *Prunus persica*), 10 g of Hong Hua (safflower, *Carthamus tinctorius*), 10 g of Gui Wei (Chinese angelica root, *Angelica sinensis*), 10 g of Fang Feng (saposhnikovia root, *Saposhnikovia divaricata*), 10 g of Chi Shao (red peony root, *Paeonia lactiflora*), 10 g of Su Mu (sappan wood, *Caesalpinia sappan*), 10 g of Pao Jia (prepared turtle shell, *Chinemys reevesii*), 10 g of Shan Nai (*Kaempferia*, *Rhizoma kaempferiae*), 10 g of Gan Jiang (dried ginger, *Zingiber officinale*), 10 g of Jiang Huang (turmeric, *Curcuma longa*), 30 g of Xiong Huang (realgar, *Arsenic sulfide*), 5 g of Bing Pian (borneol, *Dryobalanops aromatica*), and 5 g of Zhang Nao (camphor, *Cinnamomum camphora*). Use the above ingredients soaked in 1000 mL of 75% alcohol for 10 days, then, filter to obtain the medicinal wine for soaking the thread. After sufficient soaking, a ramie thread with a diameter of 0.7 mm is immersed in this medicinal liquid for more than 10 days for standby use.

**Figure 2:**
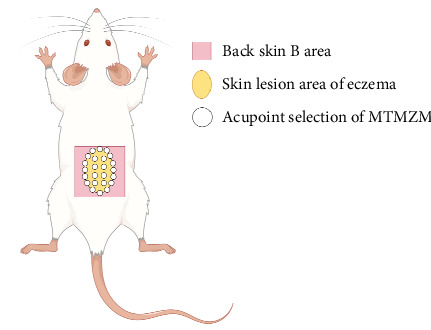
Acupoint selection of MTMZM (The image was created by the author using Figdraw, https://www.figdraw.com/).

**Figure 3:**
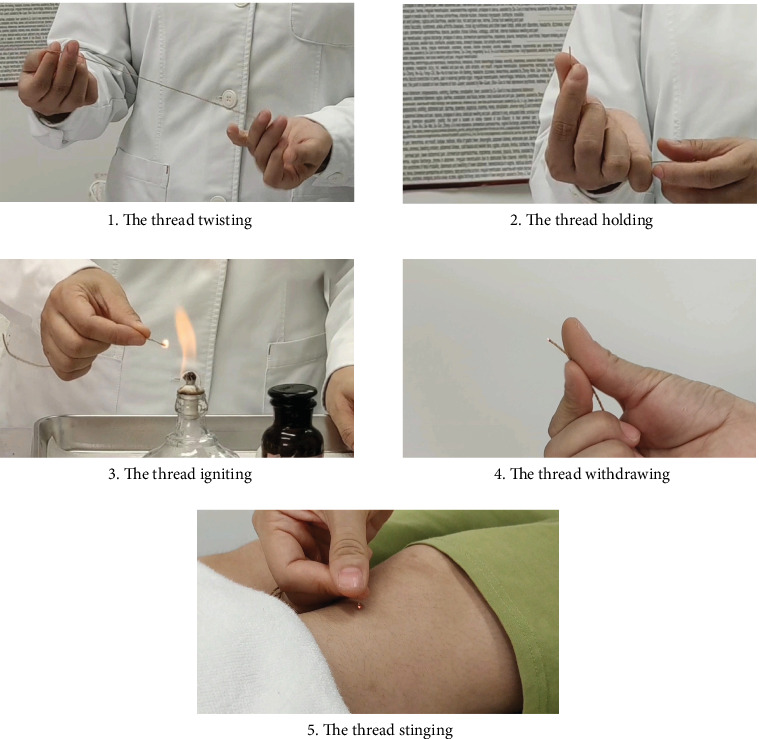
The MTMZM process in clinic.

Acupoint selection: Ashi point (equivalent to the “Jumei point” in Zhuang medicine, a group of acupoints selected along the edge and center of the lesion based on the shape of the lesion; Figure 2). Manipulation method: Two experimenters are needed to cooperate, one in charge of immobilizing the rat, and the other in charge of applying the MTMZM. To apply MTMZM, first take out the medicated thread, rub and straighten the thread tightly, hold one end of the thread with thumb and index finger, expose about 1 cm, and light the exposed thread end on the alcohol lamp. Then, the tip of burned thread should aim at the point following the flexion of wrist and thumb. When the spark in the thread becomes the bead-like ember, the thumb interphalangeal joint flexes and directly presses down the thread and makes it touched on the point [15]. Pressing the bead-like ember to extinguish is called one Zhuang [5](a single application of the medicated thread). Two Zhuang are applied per acupoint once daily, with one course of treatment lasting 1 week; the total number of courses required is two. The MTMZM process in clinic is shown in Figure 3. Normal group and model group rats were grasped and fixed at the same time and in the same way as MTMZM group, without any other intervention.

### 2.5. Material Collection

Blood was collected from the abdominal aorta after anesthesia with isoflurane, and serum was prepared and stored in aliquots. Finally, the rats were euthanized by cervical dislocation. After shaving, surgical instruments were used to sample the lesions in Area B of the rat's back skin. Normal group collected materials at the same location. The cut tissue was washed three times with precooled physiological saline and drained. Then, they were aliquoted and labeled, and stored in a −80°C refrigerator or in paraformaldehyde solution for WB detection and HE staining.

### 2.6. Observation Methods

#### 2.6.1. Eczema Severity Index (ESI)

The study referred to the scoring atopic dermatitis index (EASI) scoring standard [16] with slight modifications. The clinical symptoms of the skin of rats in each group were evaluated before, and after 1 and 2 weeks of intervention, including erythema, scale, lichenification, edema/infiltration/papules, and each symptom was scored from 0 to 3. The severity of eczema symptoms was evaluated on a scale of 0–3. In the scale, 0 means none (cannot be determined even after careful observation), 1 means mild (can only be seen after careful observation), 2 means moderate (can be seen immediately), and 3 is considered severe (very obvious). If the scores of various symptoms were not clear, 0.5 points can be recorded. Each rat was scored independently by two experimenters and the average was taken.

#### 2.6.2. Determination of STD in Eczema Model Rats

While collecting materials, a 1 cm hole punch was used to take out the diseased skin lesions and normal skin on the back of the same site, and a spiral micrometer was used to measure the skin thickness difference (STD) with normal skin and diseased skin lesions.

#### 2.6.3. HE Staining Method to Observe the Tissue Morphology of Skin

Samples were taken out from paraformaldehyde, embedded in paraffin and sectioned, deparaffinized with xylene, dehydrated with graded alcohol, stained the nucleus with hematoxylin, stained the cytoplasm with eosin, dehydrated again with graded alcohol and xylene to make it clear, dried briefly, and stained with neutral gum to cover the slide. After drying, the morphology and structure of the tissue or cells were observed through microscope.

#### 2.6.4. Western Blot to Detect the Protein Expression Levels of NF-κBp65, p-NF-κBp65, p-p38, JAK1, p-JAK1, STAT6, and p-STAT6

The skin tissue was ground to extract protein, then, the protein was homogenized and centrifuged to take the supernatant. The protein concentration was measured according to the instructions of the BCA protein quantification kit. Prepared the BCA working solution by Solution A and Solution B at a volume ratio of 50:1. Prepared the eight concentration gradients of protein standard solution required in the BCA instruction by diluting the protein standard solution using PBS. The sample was the total protein extracted by total cell protein extraction reagent. Then 20 μL protein standard solution and test sample was placed in the 96-well plates. Added 200 µL BCA working solution in each well and incubated for 30 min at 37°C after mixing. Measured and recorded the absorbance at 562 nm. Calculated the protein content based on the standard curve. Mixed with 5x loading buffer and bathed in boiled water for 5 min; after loading the sample, performed SDS-PAGE electrophoresis, cut the gel and wet transfer, the sample was soaked in BSA and placed at room temperature for about 1 h. The membrane was incubated with the primary antibody for 12 h at 4°C, washed three times with 1 × TBST, 10 min each time; then, it was incubated with the secondary antibody diluted in 1 × TBST for 1 h at 37°C, washed three times with 1x TBST, 15 min each time. After color exposure and film scanning, our team statistically analyzed the protein gray value, and calculated the content using target protein/GAPDH or target protein/β-tubulin.

#### 2.6.5. ELISA to Detect Serum IL-1β, TNF-α, and IL-4

The kit was removed from the refrigerator and placed at room temperature for 30 min to equilibrate the temperature. The blood sample was centrifuged to be tested and taken the supernatant. Following the steps of the instructions, we added samples, incubated and washed samples, at last, we used a microplate reader to test, then got the test results based on the standard curve.

### 2.7. Statistical Analysis

All data were analyzed using SPSS26.0 software. Measurement data that conformed to normal distribution were expressed as mean ± standard deviation ($\overset{―}{x}$ ± SD). Comparisons between multiple groups that conformed to normal distribution used single-factor variance. If pairwise comparisons are consistent with homogeneity of variances, LSD and Bonferroni methods are used; if not, Tamhans's T2 or Dunnett's T3 method is used. For comparisons between multiple groups that are not consistent with normal distribution, nonparametric test is used. For comparison of measurement data at multiple time points (≥3), repeated measures analysis of variance (RM-ANOVA) was used. *p*  < 0.05 was considered as a statistically significant difference.

## 3. Results

### 3.1. ESI of Rats in Each Group

**Figure 4:**
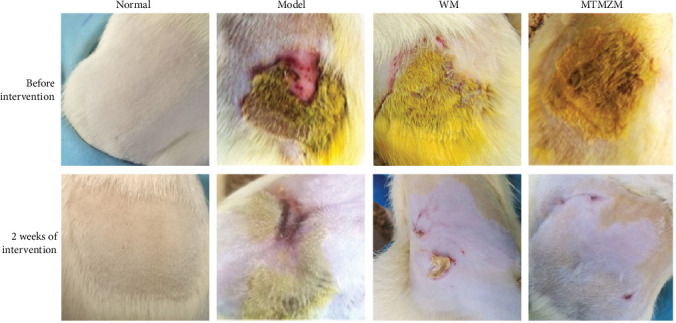
Eczema skin lesions of rats.

The changes of the lesion area are shown in Figure 4. Before the intervention, the back skin of rats in normal group was normal, with only normal grooming and no obvious scratching behavior. Eczema symptoms and signs such as erythema, papules, blisters, and erosion appeared on the skin on the back of modeled area of rats in model group, WM group, and MTMZM group, and obvious scratching occurred. After 2 weeks of intervention, the symptoms and signs of the rats in model group, WM group, and MTMZM group were better than before the intervention. There is a clear improvement in the rats in WM group and MTMZM group, the papules, vesicles, erythema, and other symptoms all dissipated to varying degrees, and their skin was obviously smoother than the rats in model group.

**Figure 5:**
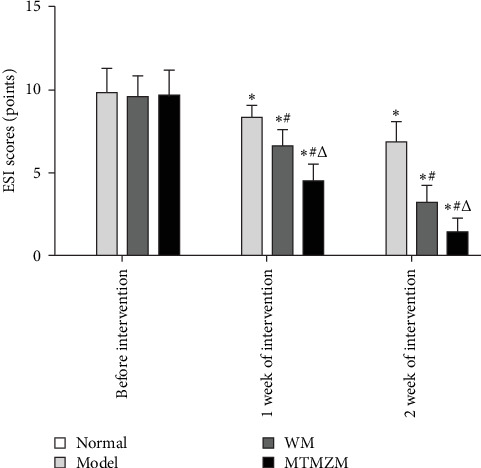
ESI scores of rats in each group. Compared with the state before intervention, *⁣*^*∗*^*p* < 0.05; compared with model group at the same time, ^#^*p* < 0.05; compared with WM group at the same time, ^△^*p* < 0.05.

ESI is shown in Figure 5. There was no statistically significant difference in the scores of rats in normal group before, and after 1 and 2 weeks of intervention (*p* > 0.05). The scores of rats in model group were lower after 1 week of intervention than before the intervention (*p* < 0.05), but there was no statistically significant difference after 2 weeks of intervention compared with 1 week of intervention (*p* > 0.05); compared with WM group at three time points, the scores all decreased (*p* < 0.05). In comparison of three time points of MTMZM group, the scores all decreased (*p* < 0.05). Before intervention, there was no statistically significant difference in ESI scores of rats in each group (*p* > 0.05). After 1 and 2 weeks of intervention: compared with the blank group, the ESI scores of model group increased (*p* < 0.05); compared with model group, the scores of rats in WM group and MTMZM group both decreased (*p* < 0.05); compared with WM group, the scores of MTMZM group decreased (*p* < 0.05).

### 3.2. STD Among Rats in Each Group

**Figure 6:**
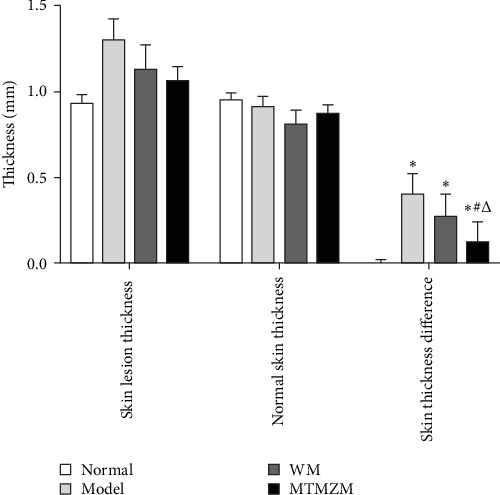
Skin thickness of rats. Compared with normal group, *⁣*^*∗*^*p* < 0.05; compared with model group, ^#^*p* < 0.05; compared with WM group, ^△^*p* < 0.05.

STDs are shown in Figure 6. Compared with normal group, STD of rats in other groups increased (*p* < 0.05); compared with model group, STD of the MTMZM group decreased (*p* < 0.05); compared with WM group, STD of MTMZM group decreased (*p* < 0.05); compared with model group, STD of MTMZM group was statistically significant (*p* < 0.05); compared with WM group, STD of MTMZM group was statistically significant (*p* < 0.05).

### 3.3. HE Staining of Skin Lesions of Rats in Each Group

**Figure 7:**
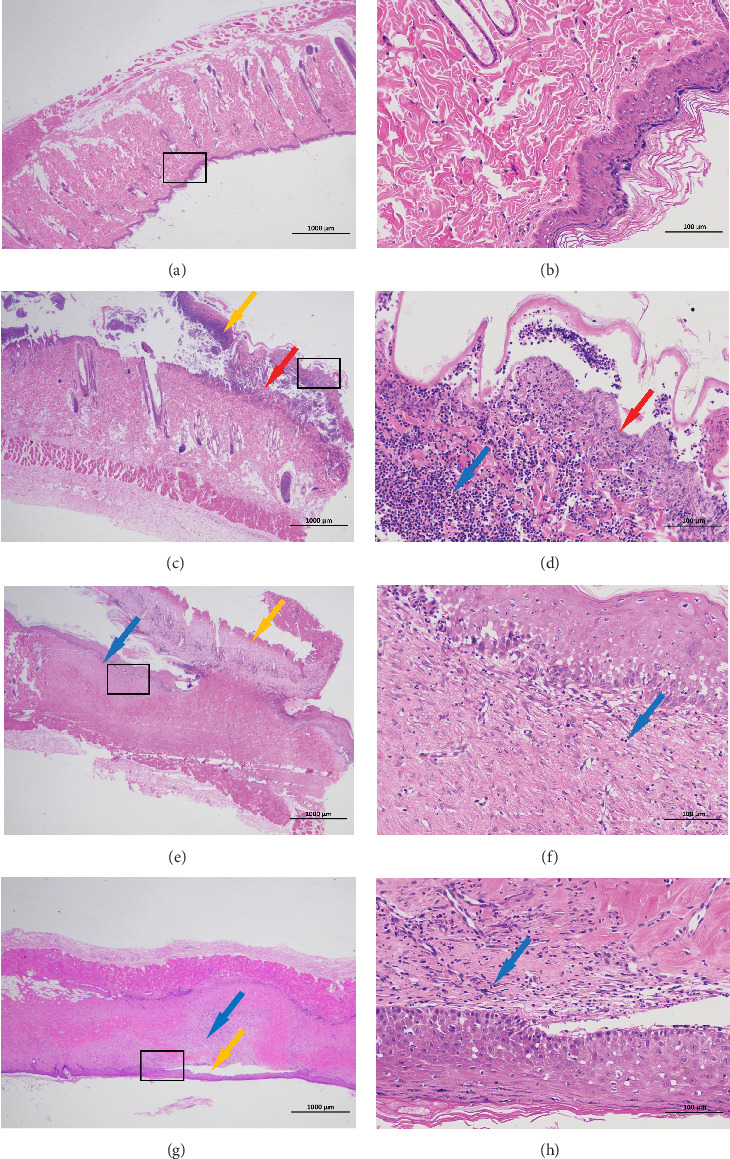
HE staining of skin lesion tissue. (A, B) Is normal group, (C, D) is model group, (E, F) is WM group, and (G, H) is MTMZM group. The scale on (A, C, E, and G) is 1000 μm, magnified by 20 times. The scale on (B, D, F, and H) is 100 μm, magnified by 200 times; black rectangle, the area where (B, D, F, and H) is located; yellow arrow, epidermal shedding or detachment; red arrow, necrotic cell debris; and blue arrow, inflammatory cell.

The results are shown in Figure 7.

The normal group exhibited an intact epidermal structure with regularly arranged, tightly packed epithelial cells. The dermis displayed abundant collagen fibers and well-developed adnexal structures (e.g., hair follicles and sebaceous glands), with no apparent inflammatory infiltrates.

In the model group, the epidermis showed marked thickening, extensive necrosis, and shedding (yellow arrows), accompanied by separation from the underlying dermis. Necrotic cellular debris was abundantly present (red arrows). The dermis revealed widespread necrosis with dense infiltration of inflammatory cells (blue arrows), but no evidence of adnexal regeneration was observed.

The WM group demonstrated partial epidermal thickening with focal necrosis and shedding (yellow arrows). A large portion of the dermis was replaced by proliferative connective tissue, with sparse inflammatory cell infiltration (blue arrows). No newly formed adnexal structures were detected.

In the MTMZM group, epidermal thickening and focal separation from the dermis were observed (yellow arrows). Extensive dermal replacement by proliferative connective tissue was noted, along with mild inflammatory cell infiltration (blue arrows). No newly formed adnexal structures were detected.

### 3.4. Protein Contents of p-p38, NF-*κ*B p65, and p-NF-*κ*B p65 in Skin Lesion Tissues of Rats in Each Group

**Figure 8:**
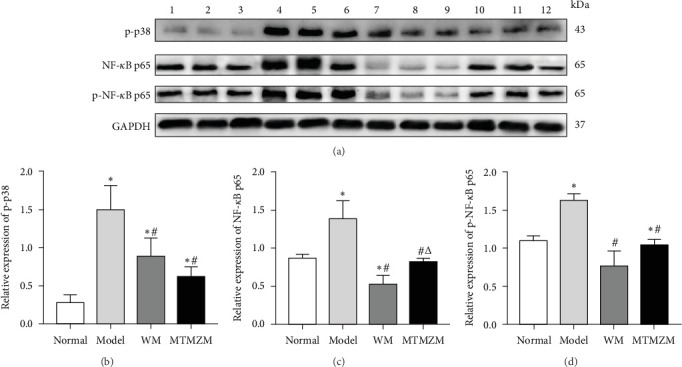
Protein contents of p-p38, NF-κB p65, and p-NF-κB p65. (A) Western blot bands of p-p38, NF-κB p65, and p-NF-κB p65: 1–3 is normal group, 4–6 is model group, 7–9 is WM group, and 10–12 is MTMZM group. (B–D) Protein expression levels of p-p38, NF-κB p65, and p-NF-κB p65. Compared with normal group, *⁣*^*∗*^*p* < 0.05; compared with model group, ^#^*p* < 0.05; compared with WM group, ^△^*p* < 0.05.

The results are shown in Figure 8.

Compared with the normal group, the p-p38, NF-κB p65, and p-NF-κB p65 of model group were increased (*p* < 0.05); p-p38 was increased (*p* < 0.05), NF-κB p65 was decreased (*p* < 0.05), and there was no significant difference in p-NF-κB p65(*p* > 0.05) of WM group; p-P38 was increased, and there was no significant difference in NF-κB p65 and p-NF-κB p65 of MTMZM group. Compared with model group, the contents of the three proteins in WM group and MTMZM group were all reduced (*p* < 0.05). Compared with WM group, the contents of p-p38 and p-NF-κB p65 has no significant change (*p* > 0.05), but the NF-κB p65 content increased (*p* < 0.05).

### 3.5. Protein Content of JAK1, p-JAK1, STAT6, and p-STAT6 in Skin Lesion Tissue of Rats in Each Group

**Figure 9:**
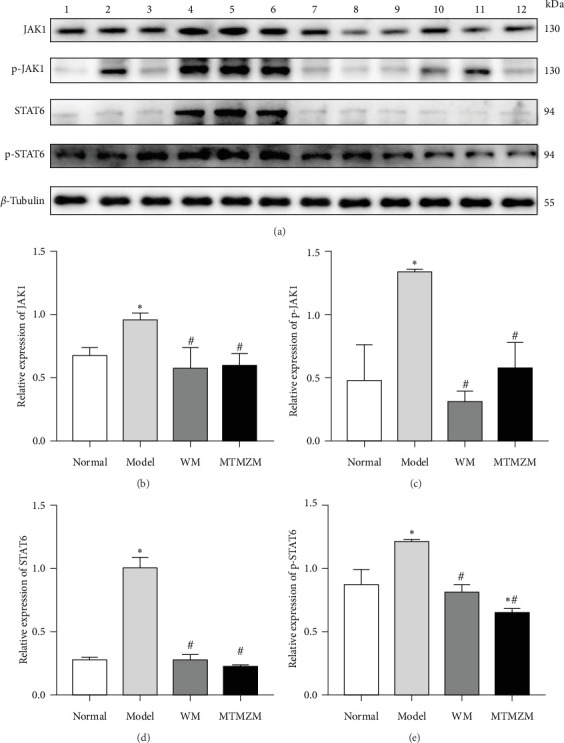
Protein contents of JAK1, p-JAK1, STAT6, and p-STAT6. (A) Western blot bands of JAK1, p-JAK1, STAT6, and p-STAT6: 1–3 is normal group, 4–6 is model group, 7–9 is WM group, and 10–12 is MTMZM group. (B–E) Protein expression levels of JAK1, p-JAK1, STAT6, and p-STAT6. Compared with normal group, *⁣*^*∗*^*p* < 0.05; compared with model group, ^#^*p* < 0.05.

As shown in Figure 9, compared with normal group, the protein contents of JAK1, p-JAK1, STAT6, and p-STAT6 in model group increased (*p* < 0.05), there was no significant change in WM group (*p* > 0.05), and the p-STAT6 in MTMZM group decreased (*p* < 0.05), and other proteins had no significant changes (*p* > 0.05); compared with model group, the JAK1, p-JAK1, STAT6, and p-STAT6 protein contents of MTMZM group and WM group decreased (*p* < 0.05); compared with WM group, there was no statistically significant difference in JAK1, p-JAK1, STAT6, and p-STAT6 protein contents in MTMZM group (*p* > 0.05).

### 3.6. Serum IL-1β, TNF-α, and IL-4 Contents of Rats in Each Group

**Figure 10:**
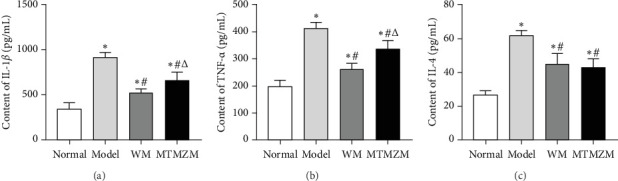
Serum IL-1β, TNF-α, and IL-4 contents of rats. (A) Content of IL-1β; (B) content of TNF-α; (C) content of IL- 4; unit: pg/mL. Compared with normal group, *⁣*^*∗*^*p* < 0.05; compared with model group, ^#^*p* < 0.05; compared with WM group,^△^*p* < 0.05.

As shown in Figure 10, compared with normal group, the contents of IL-1β, TNF-α, and IL-4 in model group were increased (*p* < 0.05); compared with model group, the contents of IL-1β, TNF-α, and IL-4 in WM group and MTMZM group were decreased (*p* < 0.05). The content of IL-1β and TNF-α in MTMZM group increased (*p* < 0.05) compared with WM group, but there was no significant difference in IL-4 content between the two groups (*p* > 0.05).

## 4. Discussion

### 4.1. Summary of Results

This study aims to explore the therapeutic effect of MTMZM on rats with eczema and its influence on P38/NF-κB and JAK1/STAT6 pathway.

The results found that eczema skin lesions exhibited erythema, papules, scabs, exudation, skin thickening, and other phenomena, leading to an increase in the ESI. The HE staining showed a large amount of inflammation and necrosis. Western blot results showed an increase in the levels of proteins associated with the P38/NF-κB and JAK1/STAT6 pathways. The ELISA results showed that there is an increase in the level of inflammatory factors. Both MTMZM and Pevisone can alleviate the symptoms of eczema, promote the recovery of skin lesions, reduce inflammation factors, and reduce the level of proteins associated with P38/NF-κB and JAK1/STAT6 pathways. However, MTMZM showed better therapeutic effects than Pevisone after 2 weeks of intervention from the symptoms of eczema.

### 4.2. Pathogenesis of Eczema and Research on P38/NF-*κ*B and JAK1/STAT6 Pathways

Existing researches mostly explore the therapeutic mechanism of eczema from aspects, such as immune system, inflammatory response, and skin barrier function. The occurrence of eczema is related to an abnormal immune system response (mainly the immune imbalance between Th1 and Th2), which produces an excessive reaction to external stimuli, leading to the occurrence and persistence of inflammatory reactions [17]. In the inflammatory reaction of eczema, a variety of inflammatory mediators are involved, such as cytokines, chemokines, leukocyte adhesion molecules, et cetera. These inflammatory mediators can cause symptoms, such as skin redness and swelling, itching, edema, and desquamation, and can further activate immune cells and inflammatory cells, forming an inflammatory cycle. Inflammation significantly affects keratinocytes in eczema. It causes abnormal proliferation and differentiation of keratinocytes, disrupts the skin barrier, induces the secretion of cytokines and chemokines, exacerbates the inflammatory response, alters the expression of antimicrobial peptides, increases the risk of infection, and ultimately aggravates the eczema condition [18].

The selection of p38/NF-κB and JAK1-STAT6 pathways was based on their established roles in eczema pathogenesis and their interconnected regulation of inflammatory cascades [19, 20]. P38, a MAPK family member, is activated by intracellular/extracellular stimuli, triggering signaling cascades. Activated P38 activates NF-κB, enabling it to enter the nucleus, regulate gene expression, and participate in biological processes. In the nucleus, activated NF-κB binds to DNA, regulating transcription of inflammation-related genes (e.g., TNF-α, IL-1β, and IL-4), triggering an inflammatory response. In eczema, TNF-α, IL-1β, and IL-4 expression increases [21], binding to receptors activates NF-κB, stimulating inflammatory mediator production (e.g., TNF-α, IL-1β, and IL-4), creating a positive feedback loop [22].

When cells are stimulated by inflammation, JAK1 phosphorylates STAT6, transferring it to the nucleus to bind DNA, activating transcription, participating in processes like cell proliferation, differentiation, apoptosis, and immune regulation [23]. Meanwhile, the JAK1-STAT6 axis is critical for Th2-polarized adaptive immunity, with IL-4 acting as a pivotal cytokine that activates this pathway to sustain chronic inflammation and IgE-mediated hypersensitivity.

IL-4, a trigger and product of the JAK1/STAT6 and P38/NF-κB pathways, is secreted mainly by Th2 cells and acts as a pivotal mediator in inflammation [24]. IL-4 activates IL-4R on the cell membrane, promoting Th2 cell proliferation and activation via the IL-4R-JAK1-STAT6 pathway. It inhibits the Th1 cytokine response via JAK-STAT, favoring Th2 immune response development, causing Th1/Th2 imbalance [25–27].

In summary, these form a self-reinforcing loop: IL-4 (via JAK1-STAT6) enhances NF-κB activation, while NF-κB upregulates IL-4 and its receptor expression. The above mechanisms can lead to chronic inflammation and persistent nonhealing of eczema. While other pathways (e.g., JNK and STAT3) contribute to eczema, p38/NF-κB and JAK1-STAT6 were prioritized due to their clinical relevance—both are validated therapeutic targets in refractory eczema, making them ideal for evaluating MTMZM's translational potential.

### 4.3. The Mechanism of MTMZM in Treating Eczema

Current researches on the treatment of eczema using MTMZM are focused primarily on clinical observations, and there are currently no research reports on the mechanism of MTMZM in the treatment of eczema through animal experiments. Clinical studies suggest that the mechanism of MTMZM in treating eczema may be related to regulating the expression of IL-2, IL-4, IL-10, and γ-IFN; therefore, it can improve the body's immunity and reduce inflammatory response [28]. Another clinical study reports the anti-inflammatory effect of MTMZM may be related to reduce the levels of TGF-β1, COX-2, IL-6, PGE2, and 5-HT [29]. However, the mechanism underlying how MTMZM inhibits these inflammatory factors remains unknown. This study found that MTMZM inhibited the P38/NF-κB and JAK1/STAT6 pathways, thereby suppressing inflammation. This discovery preliminarily reveals the mechanism of MTMZM for anti-inflammatory effects. Further support for this mechanism comes from other studies, where clinical applications and experimental research have shown that JAK-STAT inhibitors can effectively block the JAK-STAT signaling pathway, contributing to the treatment of eczema [30].

### 4.4. The Clinical Significance

MTMZM is used to treat eczema under the guidance of the unique Zhuang medicine theory of “select the first or biggest rashes as acupoints for patients with rash-induced itch.” MTMZM is manipulated on local rashes (i.e., “Jumei point” in Zhuang medicine) in the itchy area, that is, “regarding the rashes as acupoint” [31].

Pevisone cream, a widely used clinical agent for inflammatory skin conditions due to its anti-inflammatory and antiallergic properties via inhibition of inflammatory pathways [32], was selected as the positive control. The main components of Pevisone cream are triamcinolone acetonide (a glucocorticoid) and econazole (an antifungal agent). In animal models of eczema, the SD rats were maintained in a SPF environment and eczema was induced using DNCB, a chemical sensitizer known to elicit T cell-mediated inflammatory response without fungal involvement. Given the absence of fungal colonization in this model, the antifungal properties of econazole nitrate are unlikely to contribute therapeutic effects. This means that triamcinolone acetonide serves as the primary active component—a potent glucocorticoid with strong and sustained anti-inflammatory and antiallergic properties. Therefore, therapeutic effect of Pevisone cream is primarily attributed to the anti-inflammatory action of triamcinolone acetonide.

Experimental findings indicate that the therapeutic effect of MTMZM is better than applying Pevisone cream on skin lesions. This discovery, which is largely consistent with the previous clinical trials conducted by our research team [6, 7], provides clinicians with a new and potentially more effective treatment strategy, especially for patients who are insensitive to drug treatments or seek alternative therapies. It also contributes to promoting the integration of Chinese medicine and WM in the treatment of skin diseases such as eczema, providing more scientific evidence and practical experience for the combined treatment approach of Chinese and WM. Currently, the research on MTMZM is still in its preliminary stage, and further experimental studies need to be conducted to further explore its mechanism, safety, and optimization of treatment protocols.

### 4.5. Research Limitations and Prospects

This study still has certain limitations: (a) How are these two pathways directly affected by MTMZM? Is it the burning thermal effect or the effect of the medicine? Further mass spectrometry analysis and experiments are needed to confirm. (b) The pathogenesis and treatment mechanisms of eczema are quite complex, and there may be interactions between different signaling pathways. This study only investigated two of these pathways and did not directly examine their interactions. The major pathway changes after MTMZM treatment can be verified by RNA sequencing. (c) Due to various limitations, the behavior of scratching an itch has not been observed well. Artificial intelligence recognition technology needs to be utilized to observe this behavior in a more objective manner. Above these are among the key research directions that research team will prioritize in the future.

## 5. Summary

In summary, this study used observation recording, HE staining, WB, and ELISA to study the efficacy and mechanism of MTMZM in the treatment of eczema. The results show that MTMZM can effectively relieve eczema skin lesions, which may be related to the inhibition of p38/NF-κB and JAK1-STAT6 pathways, while clinical translation requires further validation.

This study, rooted in the principles of traditional Zhuang medicine, provides evidence that MTMZM may serve as a viable adjunctive therapy to conventional dermatological treatments. While not intended to replace standard medical approaches, MTMZM demonstrates favorable clinical outcomes in terms of lesion resolution, safety, and cost-effectiveness, particularly in resource-limited or community-based healthcare settings.

Importantly, although MTMZM has been passed down through generations as a traditional therapy, animal models were employed in this study to better elucidate its underlying mechanisms, such as anti-inflammatory modulation via the p38/NF-κB and JAK1-STAT6 pathways. This mechanistic insight complements its empirical use and supports further translational research.

In conclusion, the integration of MTMZM as a complementary approach merits further clinical investigation, particularly through controlled human studies, to establish its efficacy, safety, and standardization in the context of evidence-based medicine.

## Data Availability

The dataset can be obtained from the corresponding author upon reasonable request.
